# Integrating Machine Learning with Multi-Omics Technologies in Geroscience: Towards Personalized Medicine

**DOI:** 10.3390/jpm14090931

**Published:** 2024-08-31

**Authors:** Nikolaos Theodorakis, Georgios Feretzakis, Lazaros Tzelves, Evgenia Paxinou, Christos Hitas, Georgia Vamvakou, Vassilios S. Verykios, Maria Nikolaou

**Affiliations:** 1 Department of Cardiology & 65+ Clinic, Amalia Fleming General Hospital, 14, 25th Martiou Str., 15127 Melissia, Greece; n.theodorakis@flemig-hospital.gr (N.T.); ch.chitas@flemig-hospital.gr (C.H.); g.vamvakou@flemig-hospital.gr (G.V.); m.nikolaou@flemig-hospital.gr (M.N.); 2 School of Medicine, National and Kapodistrian University of Athens, 75 Mikras Asias, 11527 Athens, Greece; 3 School of Science and Technology, Hellenic Open University, 18 Aristotelous Str., 26335 Patras, Greece; georgios.feretzakis@ac.eap.gr (G.F.); paxinou.evgenia@ac.eap.gr (E.P.); 4 2nd Department of Urology, Sismanoglio General Hospital, Sismanogliou 37, National and Kapodistrian University of Athens, 15126 Athens, Greece; laztz@med.uoa.gr

**Keywords:** machine learning, multi-omics technologies, aging research, geroscience, hallmarks of aging, personalized medicine

## Abstract

Aging is a fundamental biological process characterized by a progressive decline in physiological functions and an increased susceptibility to diseases. Understanding aging at the molecular level is crucial for developing interventions that could delay or reverse its effects. This review explores the integration of machine learning (ML) with multi-omics technologies—including genomics, transcriptomics, epigenomics, proteomics, and metabolomics—in studying the molecular hallmarks of aging to develop personalized medicine interventions. These hallmarks include genomic instability, telomere attrition, epigenetic alterations, loss of proteostasis, disabled macroautophagy, deregulated nutrient sensing, mitochondrial dysfunction, cellular senescence, stem cell exhaustion, altered intercellular communication, chronic inflammation, and dysbiosis. Using ML to analyze big and complex datasets helps uncover detailed molecular interactions and pathways that play a role in aging. The advances of ML can facilitate the discovery of biomarkers and therapeutic targets, offering insights into personalized anti-aging strategies. With these developments, the future points toward a better understanding of the aging process, aiming ultimately to promote healthy aging and extend life expectancy.

## 1. Introduction

### 1.1. Background on Aging and Its Medical Significance

Aging is an inevitable biological process marked by a progressive decline in cellular and physiological functions. This decline is associated with increased vulnerability to a range of chronic diseases, including cardiovascular diseases (CVDs), neurodegenerative disorders, diabetes, and cancers [1]. Aging not only affects the health and quality of life of individuals but also poses significant socioeconomic challenges due to rising healthcare costs and the need for long-term care [2].

The study of aging, or geroscience, aims to understand the underlying mechanisms that drive this process. The primary focus of geroscience is to identify strategies to mitigate the complications of aging, thereby extending the period of healthy disease-free life, known as health span and overall lifespan [1]. This pursuit has led to the identification of a set of biological processes termed the hallmarks of aging. These hallmarks represent the common molecular mechanisms that mediate the aging process across different species and include genomic instability, telomere attrition, epigenetic alterations, loss of proteostasis, disabled macroautophagy, deregulated nutrient sensing, mitochondrial dysfunction, cellular senescence, stem cell exhaustion, altered intercellular communication, chronic inflammation, and dysbiosis [2].

### 1.2. Overview of Multi-Omics Data

The advances in omics technologies have revolutionized biological research, providing comprehensive datasets that capture different layers of biological information. Multi-omics approaches, which integrate data from various omics technologies, offer a holistic view of the molecular mechanisms underlying complex biological processes like aging. Genomics focuses on the study of the entire genome, including genes and regulatory elements. It provides insights into genetic predispositions and mutations that contribute to aging and age-related diseases [3]. Epigenomics examines modifications to the genetic material that affect gene expression without altering the DNA sequence. These include DNA methylation, histone modification, and non-coding RNA molecules. Epigenetic changes are crucial in regulating gene expression and are significantly impacted by aging [4]. Transcriptomics involves the study of RNA transcripts produced by the genome. It reflects gene expression and provides information on how genetic information is translated into functional proteins. Transcriptomic analysis helps in understanding gene regulation and its alterations during aging [5]. Proteomics is the large-scale study of proteins, including their three-dimensional structure, function, and interactions. Proteins are the primary effectors of cellular functions, and proteomic analyses reveal changes in protein expression, post-translational modifications, and interactions that occur in the context of aging [6]. Metabolomics is the comprehensive analysis of metabolites, the small molecules involved in metabolic pathways. Metabolomic profiling provides insights into the biochemical activities and metabolic state of the cell and therefore organism, which can reveal the metabolic shifts associated with aging [7].

### 1.3. Importance of Machine Learning in Modern Biomedical Research

Machine learning (ML) has become an indispensable tool in biomedical research, particularly for handling and interpreting complex high-dimensional datasets generated by omics technologies. The main strength of ML is its ability to identify patterns within data and use them to make predictions or classifications.

ML algorithms are adept at managing large-scale and heterogeneous data, identifying intricate patterns, and extracting meaningful insights. This capability is particularly useful in integrating multi-omics data to understand the multifaceted nature of aging [8].

ML has been applied to various aspects of aging research, including the identification of biomarkers, prediction of disease onset and progression, and the development of personalized treatment strategies. For instance, ML models can predict biological age based on omics data, identify key molecular drivers of aging, and suggest potential therapeutic targets [9].

### 1.4. Objectives

This review aims to provide a comprehensive overview of how integrating multi-omics data with ML techniques can enhance our understanding of the hallmarks of aging. We will discuss the role of different omics technologies in studying each hallmark of aging. Furthermore, we will explore how ML models can be applied to predict and analyze age-related changes. Additionally, we will highlight case studies and applications that demonstrate the potential of this integrative approach. Moreover, we will report the challenges and limitations in the field. Finally, we will suggest future directions for advancing aging research and developing personalized strategies to extend health span and lifespan.

## 2. Hallmarks of Aging and Multi-Omics Data Integration

### 2.1. Genomic Instability

#### 2.1.1. Role of Genetic Variations and Mutations in Aging

Genomic instability is a primary hallmark of aging, characterized by an increased frequency of mutations and structural alterations in the DNA. These mutations arise from DNA replication errors, environmental insults, and defective DNA repair mechanisms. Key sources of genomic instability include the accumulation of reactive oxygen species (ROS), which cause oxidative damage to DNA, and the decline in the efficiency of DNA repair pathways such as base excision repair (BER), nucleotide excision repair (NER), mismatch repair (MMR), and double-strand break repair mechanisms including homologous recombination and non-homologous end joining [10]. Studies have shown that aged cells exhibit higher levels of DNA damage and chromosomal aberrations, which can drive oncogenesis and cellular dysfunction. For instance, increased rates of somatic mutations in mitochondrial DNA (mtDNA) have been linked to reduced cellular respiration and increased oxidative stress, further exacerbating genomic instability [11]. Experimental evidence from model organisms, such as mice with impaired DNA repair capabilities, demonstrates accelerated aging phenotypes, underscoring the critical role of genomic integrity in longevity [12].

#### 2.1.2. Machine Learning Models to Predict Genomic Instability and Its Effects

ML models are particularly useful in predicting the effects of genomic instability by analyzing large-scale genomic datasets. Supervised learning ML algorithms, like support vector machines (SVMs) and neural networks, can be trained on labeled genomic data to classify mutations as benign or pathogenic, which is critical for understanding which mutations contribute to aging and age-related diseases. Unsupervised learning techniques like clustering can identify novel mutational signatures that may not be evident through traditional analysis. These signatures can be associated with aging, leading to insights into the genomic changes that accumulate over time [13].

In a specific example, Xu et al. developed a deep learning model to predict genomic instability from histopathology slides. When applied to 1010 patients with breast cancer, the model accurately classified chromosomal instability status with with 81.2% sensitivity and 68.7% specificity in the test set [14].

### 2.2. Telomere Attrition

#### 2.2.1. Mechanisms of Telomere Shortening and Its Impact on Cellular Aging

Telomeres are repetitive nucleotide sequences at the ends of chromosomes that protect them from degradation and prevent chromosomal fusions. With each cell division, telomeres shorten due to the end-replication problem, where DNA polymerase cannot fully replicate the ends of linear chromosomes. Critically short telomeres trigger a DNA damage response, leading to cellular senescence or apoptosis. The enzyme telomerase can elongate telomeres by adding telomeric repeats; however, its expression is limited in most somatic cells. Telomere shortening has been implicated in the aging of high-turnover tissues, such as hematopoietic and epithelial cells. Experimental studies have demonstrated that telomere dysfunction activates the p53 pathway, leading to cell cycle arrest and senescence. In contrast, overexpression of telomerase in mouse models extends lifespan and delays the onset of age-related pathologies, highlighting the therapeutic potential of targeting telomere attrition to combat aging [15].

#### 2.2.2. Integrative Approaches to Study Telomere Dynamics Using Multi-Omics Data

ML models can integrate multi-omics datasets to analyze key regulators of telomere dynamics and predict the effects of telomere shortening on cellular function. For example, ML algorithms can predict the onset of cellular senescence based on the combination of telomere length, gene expression profiles, and protein interaction networks. Furthermore, ML can help stratify individuals based on their risk of telomere attrition-related conditions by predicting telomere dynamics and linking them to specific age-related diseases [16].

In a specific example, Zhang et al. developed an ML approach to predict telomere length from whole-genome sequencing data. They used a random forest model trained on features extracted from sequencing reads that mapped to telomeric regions. The model was trained on and validated by data from the 1000 Genomes Project and showed high accuracy in predicting telomere length. This method provides a cost-effective alternative to experimental telomere length measurement and could be valuable for large-scale studies on aging and age-related diseases [17].

### 2.3. Epigenetic Alterations

#### 2.3.1. Age-Associated Changes in Epigenetic Markers

Epigenetic changes, including DNA methylation, histone modifications, and chromatin remodeling, play a crucial role in regulating gene expression. Age-associated epigenetic alterations can disrupt normal gene regulatory networks, leading to changes in cellular function and contributing to aging and age-related diseases. For instance, global DNA hypomethylation and site-specific hypermethylation of CpG islands in gene promoters have been observed in aged tissues. These changes can lead to the silencing of tumor suppressor genes and the activation of oncogenes, promoting cancer development. Histone modifications, such as the loss of histone H3 lysine 27 trimethylation (H3K27me3), are also associated with aging and contribute to the deregulation of gene expression. Research using epigenetic clocks, which measure biological age based on DNA methylation patterns, has shown that these clocks can predict lifespan and age-related disease risk, demonstrating the significance of epigenetic changes in aging [18].

#### 2.3.2. Machine Learning Techniques to Identify and Predict Epigenetic Alterations

Supervised ML models, such as linear regression and deep neural networks, can be trained to predict biological age based on DNA methylation patterns, often referred to as epigenetic clocks. These models are key in estimating an individual’s biological age versus chronological age. Furthermore, ML can identify age-associated epigenetic markers by clustering samples based on DNA methylation and histone modification data, providing a clearer picture of how epigenetic alterations drive aging [19].

In a specific example, Higgins-Chen et al. introduced a new epigenetic clock using deep neural networks, addressing key limitations of previous clocks. Their model, called DunedinPACE, predicts the pace of aging from DNA methylation data. Unlike previous clocks that estimate biological age, DunedinPACE focuses on the rate of aging, providing a more dynamic measure. The model was trained on longitudinal data and validated across multiple cohorts, showing improved reliability and sensitivity to biological aging interventions [20].

### 2.4. Loss of Proteostasis

#### 2.4.1. Disruption in Protein Homeostasis and Its Implications

Proteostasis, or protein homeostasis, involves the maintenance of the correct concentration, conformation, and localization of proteins within cells. Disruption of proteostasis is a hallmark of aging and is linked to various age-related diseases, including neurodegenerative disorders like Alzheimer’s and Parkinson’s diseases. The accumulation of misfolded and aggregated proteins, which escape the cellular quality control systems such as the ubiquitin–proteasome system and autophagy, can lead to cellular toxicity and dysfunction. Experimental models have shown that enhancing proteostasis pathways, such as upregulating chaperone proteins or activating autophagy, can ameliorate age-related proteotoxicity and extend lifespan. For example, caloric restriction and pharmacological activation of autophagy have been shown to reduce protein aggregation and improve cellular function in aging models, highlighting potential therapeutic strategies for restoring proteostasis [21].

#### 2.4.2. Omics Data to Study Proteostasis and Machine Learning Models to Predict Proteostasis-Related Disorders

ML models, especially deep learning approaches, can analyze large-scale proteomic data to identify patterns of protein misfolding, aggregation, and degradation that are characteristic of aging cells. Supervised learning can predict the likelihood of proteostasis-related disorders, such as neurodegenerative diseases, by analyzing protein expression profiles and identifying key proteins that lose stability with age [22,23].

In a specific example, Eshari et al. developed an logistic regression model to predict protein aggregation propensity from sequence data. Their model outperformed existing methods in predicting aggregation-prone regions in proteins. This approach could be valuable for understanding protein misfolding in age-related neurodegenerative diseases and for designing therapeutic interventions [24].

### 2.5. Disabled Macroautophagy

#### 2.5.1. Importance of Autophagy in Aging and Age-Related Diseases

Macroautophagy, commonly referred to as autophagy, is a cellular process that degrades and recycles damaged organelles, proteins, and other macromolecules. Autophagy is essential for maintaining cellular homeostasis and responding to stress. Impairment of autophagy is associated with aging and age-related diseases, including neurodegenerative disorders and cancers. Studies have shown that autophagy declines with age, leading to the accumulation of damaged cellular components and increased cellular stress. Genetic and pharmacological interventions that enhance autophagy have been shown to extend lifespan and delay the onset of age-related pathologies in model organisms. For instance, activation of the autophagy-related gene Atg5 in mice enhances autophagic activity and promotes healthy aging, suggesting that targeting autophagy pathways could be a viable strategy to combat aging [25].

#### 2.5.2. Combining Multi-Omics Data to Understand and Enhance Autophagy Processes

ML can integrate transcriptomic, proteomic, and metabolomic data to predict the levels of autophagic activity in cells. This can help identify individuals at risk for age-related diseases due to impaired autophagy. By analyzing multi-omics data, ML models can identify the key regulators of autophagy and suggest potential therapeutic targets to restore autophagic processes in aging cells [26].

In a specific example, Dong et al. explored autophagy-related biomarkers in peripheral blood for diagnosing rheumatoid arthritis using machine learning. Researchers identified 25 differentially expressed autophagy-related genes (DE-ARGs) and used algorithms like random forest and LASSO to pinpoint three key biomarkers that showed strong diagnostic value across multiple validation cohorts [27].

### 2.6. Deregulated Nutrient Sensing

#### 2.6.1. Impact of Nutrient Sensing Pathways on Aging

Nutrient sensing pathways, such as the insulin/IGF-1 signaling (IIS) pathway, mechanistic target of rapamycin (mTOR), AMP-activated protein kinase (AMPK), and sirtuins, play critical roles in regulating metabolism, growth, and aging. Deregulation of these pathways can lead to metabolic imbalances and contribute to aging and age-related diseases. For example, the mTOR pathway, which promotes anabolic processes and inhibits catabolic processes like autophagy, is overactive in many age-related diseases. In contrast, activation of AMPK and sirtuins, which promote catabolic processes and enhance stress resistance, is associated with increased lifespan and improved metabolic health. Experimental evidence from model organisms, such as caloric restriction and pharmacological inhibition of mTOR, has demonstrated that the modulation of nutrient sensing pathways can extend lifespan and delay the onset of age-related diseases, highlighting the therapeutic potential of targeting these pathways [28].

#### 2.6.2. Integrative Models to Study the Regulation and Deregulation of These Pathways

Supervised ML algorithms can predict the activation state of nutrient sensing pathways (like mTOR, AMPK, and sirtuins) based on gene expression and proteomic data, aiding in the understanding of how these pathways contribute to aging. Unsupervised learning techniques can identify novel regulatory networks within nutrient sensing pathways, providing deeper insights into how these pathways are deregulated in aging [29].

In a specific example, Drewe et al. used machine learning models to identify AMPK activators, which play a crucial role in regulating cellular metabolism and are beneficial in treating diseases like diabetes and cancer. Various algorithms, including random forest and deep neural networks, were tested and showed high accuracy in distinguishing activators from controls. The models are suitable for screening potential AMPK activators, including natural compounds, and can guide further in vitro testing to identify promising candidates for drug development [30].

### 2.7. Mitochondrial Dysfunction

#### 2.7.1. Role of Mitochondrial Function and Dysfunction in Aging

Mitochondria are the powerhouses of the cell, responsible for producing ATP through oxidative phosphorylation. Mitochondrial dysfunction is a hallmark of aging and is characterized by impaired energy production, increased production of reactive oxygen species (ROS), and mitochondrial DNA (mtDNA) mutations. These changes contribute to cellular damage and the decline in physiological functions observed in aging. Studies have shown that mitochondrial biogenesis and dynamics, including fission and fusion processes, decline with age, leading to the accumulation of dysfunctional mitochondria. Interventions that enhance mitochondrial function, such as caloric restriction and mitochondrial-targeted antioxidants, have been shown to extend lifespan and improve metabolic health in model organisms, suggesting that targeting mitochondrial dysfunction could be a viable strategy to combat aging [31].

#### 2.7.2. Multi-Omics Approaches to Study Mitochondrial Health and Predictive Models

ML models can predict mitochondrial dysfunction by integrating various omics data (genomic, transcriptomic, proteomic) and identifying patterns associated with impaired mitochondrial function. ML can also reveal how mitochondrial dysfunction interacts with other cellular processes, such as oxidative stress, by analyzing complex data interactions and predicting the downstream effects [32].

In a specific example, Qin developed the mitochondrial programmed cell death index as a strong prognostic tool for lower-grade glioma using ML on data from 1467 patients. This index, based on 18 key genes, outperformed existing clinical models in predicting patient outcomes. Patients with high index had worse survival, increased immune activity, and higher tumor mutation burden, suggesting its potential in guiding personalized treatment and immunotherapy [33].

### 2.8. Cellular Senescence

#### 2.8.1. Mechanisms and Consequences of Cellular Senescence

Cellular senescence is a state of permanent cell cycle arrest that occurs in response to various stressors, such as DNA damage, oxidative stress, and telomere shortening. Senescent cells contribute to aging and age-related diseases by secreting pro-inflammatory factors and other molecules that disrupt tissue function, known as the senescence-associated secretory phenotype (SASP). Studies have shown that the accumulation of senescent cells in tissues leads to chronic inflammation, tissue dysfunction, and the promotion of age-related diseases such as osteoarthritis and atherosclerosis. Experimental evidence from model organisms has demonstrated that the selective elimination of senescent cells, known as senolysis, can delay aging and extend lifespan, highlighting the therapeutic potential of targeting cellular senescence [34].

#### 2.8.2. Using Omics Data and Machine Learning to Identify Senescent Cells and Develop Interventions

Supervised ML models can classify cells based on their senescence status by analyzing the expression of cell cycle inhibitors and SASP factors, allowing for the identification of senescent cells within tissues. Furthermore, ML can also model the composition and dynamics of the SASP, providing insights into how these factors contribute to tissue dysfunction and aging [35].

In a specific example, Tuttle et al. conducted a systematic review and meta-analysis of cellular senescence markers across various human tissues. While not directly applying ML, their comprehensive analysis provides a foundation for future ML studies on cellular senescence. They synthesized data from multiple studies to identify consistent markers of senescence across different tissues and age groups. This work is crucial for developing ML models to predict cellular senescence and its impact on aging [36].

### 2.9. Stem Cell Exhaustion

#### 2.9.1. Decline in Stem Cell Function with Age

Stem cells are essential for tissue regeneration and repair. However, their function declines with age, leading to impaired tissue maintenance and regeneration. Stem cell exhaustion is characterized by a reduction in the number and function of stem cells, which contributes to the decline in tissue homeostasis and repair capacity observed in aging. Studies have shown that aged stem cells exhibit increased DNA damage, epigenetic alterations, and a decline in their regenerative potential. Interventions that rejuvenate aged stem cells, such as the activation of signaling pathways that promote stem cell self-renewal or the transplantation of young stem cells, have been shown to improve tissue regeneration and extend lifespan in model organisms, suggesting that targeting stem cell exhaustion could be a viable strategy to combat aging [37].

#### 2.9.2. Integrative Approaches to Study Stem Cell Biology and Predictive Models

ML models can predict the likelihood of stem cell exhaustion by analyzing gene expression and protein interaction networks, helping to identify key factors that lead to the decline in stem cell function with age. These models can also help in planning interventions by predicting the effects of potential treatments on stem cell function, guiding strategies to rejuvenate aged stem cells [38].

In a specific example, Barardo et al. developed an ML model to predict lifespan-extending compounds, which could potentially address stem cell exhaustion. They used a random forest classifier trained on various molecular and chemical features to predict compounds that might extend lifespan. While not directly focused on stem cells, many lifespan-extending compounds work by preserving stem cell function. This approach demonstrates how ML can be used to identify potential interventions for age-related stem cell exhaustion [39].

### 2.10. Altered Intercellular Communication

#### 2.10.1. Changes in Cell Signaling and Communication in Aging

Cell signaling and communication are critical for maintaining tissue homeostasis. Altered intercellular communication is a hallmark of aging and can lead to disrupted tissue function and inflammation. For example, age-related changes in the immune system, known as immunosenescence, can impair the body’s ability to respond to infections and repair tissue damage. Studies have shown that the aged immune system exhibits a decline in the production of cytokines and chemokines, leading to a reduced ability to mount an effective immune response. Interventions that restore normal intercellular communication, such as the administration of cytokines or the transplantation of young immune cells, have been shown to improve immune function and extend lifespan in model organisms, suggesting that targeting altered intercellular communication could be a viable strategy to combat aging [40].

#### 2.10.2. Omics Data to Study Intercellular Communication and Machine Learning Models to Predict Alterations

ML can classify cells based on their signaling activity by integrating transcriptomic and proteomic data, helping to identify disruptions in cell communication that contribute to aging. By revealing how altered intercellular communication interacts with processes like inflammation, ML models can guide the development of therapies aimed at restoring normal cell signaling [41].

In a specific example, Wang et al. developed iTALK, an R package that uses ML algorithms to analyze single-cell RNA-seq data and predict cell–cell communication networks. While not specifically focused on aging, this tool has significant potential for studying how intercellular communication changes with age. iTALK can identify ligand-receptor pairs and visualize communication networks, providing insights into how cellular interactions are altered in aging tissues [42].

### 2.11. Chronic Inflammation

#### 2.11.1. Role of Chronic Inflammation in Aging and Age-Related Diseases

Chronic inflammation, also known as “inflammaging”, is a characteristic of aging and is linked to various age-related diseases, including CVD, diabetes mellitus, and neurodegenerative disorders. Persistent low-grade inflammation can contribute to tissue damage and dysfunction, exacerbating the aging process. Studies have shown that aged tissues exhibit increased levels of pro-inflammatory cytokines and a decline in anti-inflammatory signaling pathways. Experimental evidence from model organisms has demonstrated that the inhibition of pro-inflammatory pathways or the activation of anti-inflammatory pathways can extend lifespan and improve health span, highlighting the therapeutic potential of targeting chronic inflammation [43].

#### 2.11.2. Combining Multi-Omics Data to Study Inflammation and Predictive Models

ML models can classify cells based on their inflammatory activity by analyzing gene expression profiles and proteomic data, identifying those with high levels of chronic inflammation (inflammaging). These models can also reveal how chronic inflammation interacts with other cellular processes, such as oxidative stress, offering insights into potential anti-inflammatory interventions [44].

In a specific example, Bobrov et al. developed PhotoAgeClock, a deep learning model that predicts biological age from facial images. While not directly measuring inflammation, this non-invasive approach can capture signs of aging that are often related to chronic inflammation. The model was trained on a large dataset of facial images and showed high correlation with chronological age. This study demonstrates how ML can be used to develop non-invasive biomarkers of aging, which could be valuable for studying the effects of chronic inflammation on aging [45].

### 2.12. Dysbiosis

#### 2.12.1. Age-Related Changes in the Microbiome

Dysbiosis refers to changes in the composition and function of the microbiome. The microbiome, which consists of trillions of microorganisms residing in the gut and other tissues, plays a critical role in regulating metabolism, immunity, and overall health. Alterations in the microbiome can influence various aspects of health and contribute to aging and age-related diseases. Studies have shown that aged individuals exhibit a decline in microbial diversity and an increase in the abundance of pathogenic bacteria. Experimental evidence from model organisms has demonstrated that the restoration of a healthy microbiome, through the administration of probiotics or fecal microbiota transplantation, can improve metabolic health and extend lifespan, suggesting that targeting dysbiosis could be a viable strategy to combat aging [46].

#### 2.12.2. Integrative Approaches to Study the Microbiome and Machine Learning Models to Predict Dysbiosis

ML can classify microbiome samples based on their age-related profiles, using metagenomic and metabolomic data to identify signatures of dysbiosis that contribute to aging. By integrating omics data, ML models can reveal how dysbiosis interacts with host processes, such as metabolism and immune function, guiding the development of microbiome-targeted interventions [46].

In a specific example, Wilmanski et al. (2021) used ML to analyze microbiome data and predict biological age. They developed a random forest model trained on gut microbiome composition data from a large cohort of individuals. The model identified specific microbial features associated with healthy aging and longevity. The study demonstrated how ML can be used to understand the complex relationship between the gut microbiome and aging, providing insights into potential interventions to promote healthy aging through microbiome modulation [47].

A summary of the aging hallmarks, their molecular mechanisms, and potential interventions is presented in Table 1.

## 3. Machine Learning Techniques in Medicine

### 3.1. Overview of Machine Learning Algorithms

ML encompasses a variety of algorithms that can be broadly categorized into supervised, unsupervised, and reinforcement learning.

In supervised learning, algorithms learn from labeled training data to make predictions or classifications. Examples include linear regression, decision trees, random forests, support vector machines (SVMs), and neural networks. In supervised learning, the algorithm is trained on input–output pairs, allowing it to predict outcomes for new unseen data [68].

In unsupervised learning, algorithms identify patterns or structures within unlabeled data. Examples include clustering algorithms like k-means, hierarchical clustering, and dimensionality reduction techniques like principal component analysis (PCA) and t-distributed stochastic neighbor embedding (t-SNE). Unsupervised learning is useful for discovering hidden patterns and structures in data without prior knowledge of the outcomes [69].

In reinforcement learning, algorithms learn by interacting with an environment to maximize cumulative rewards. This approach is often used in robotics, game playing, and autonomous systems. Reinforcement learning involves learning a policy that maps states to actions to achieve the highest reward [70].

In deep learning, algorithms use neural networks with multiple layers (hence “deep”) to model complex patterns in large datasets. Examples include convolutional neural networks (CNNs) for image data and recurrent neural networks (RNNs) for sequential data. Deep learning models are particularly powerful for integrating multi-omics data, identifying intricate patterns, and making accurate predictions in biomedical research [71].

In biomedical research, these algorithms can be applied to various tasks, including disease diagnosis, treatment prediction, and biomarker discovery. For example, supervised learning algorithms can predict patient outcomes based on clinical and omics data, while unsupervised learning techniques can identify subgroups of patients with distinct molecular profiles [8]. Deep learning models can integrate various types of omics data to provide comprehensive insights into complex biological processes and enhance predictive accuracy.

### 3.2. Challenges in Handling Biomedical Data

Biomedical data present several challenges, including data heterogeneity, integration, and quality.

Data heterogeneity. Biomedical data come from various sources and formats, such as genomic sequences, imaging data, and electronic health records. Integrating these diverse datasets requires sophisticated data preprocessing and normalization techniques. For example, genomic data may need to be aligned and variant-called, while imaging data may require segmentation and feature extraction [72].

Data integration. Combining multi-omics data requires careful consideration of the different scales and types of data involved. Advanced computational models and algorithms are needed to effectively integrate these datasets. For instance, multi-omics integration may involve combining high-dimensional genomic data with lower-dimensional clinical data, requiring techniques like matrix factorization or network-based approaches [73].

Data quality. Biomedical data can be noisy, incomplete, or biased. Ensuring data quality is crucial for the development of accurate and reliable ML models. Data preprocessing steps, such as normalization, imputation, and outlier detection, are essential for improving data quality. Additionally, techniques like cross-validation and bootstrapping can help assess model robustness and reliability [71].

Solutions for these challenges include preprocessing techniques such as normalization and imputation, feature selection methods to reduce dimensionality, and robust model evaluation strategies to assess model performance. For example, normalization methods can adjust for batch effects in omics data, while feature selection techniques can identify the most informative variables for model building. Cross-validation and external validation on independent datasets are crucial for assessing model generalizability and preventing overfitting [69].

### 3.3. Advances in Machine Learning for Multi-Omics Data Integration

Recent advances in ML have enabled the more sophisticated integration of multi-omics data. Deep learning techniques, such as convolutional neural networks (CNNs) and recurrent neural networks (RNNs), have shown promise in analyzing complex biomedical data. These models can automatically learn hierarchical representations from raw data, making them suitable for integrating multi-omics data [71].

Graph-based approaches, such as graph neural networks (GNNs), can capture the relationships between different omics layers and provide a more holistic view of biological systems. These methods can model interactions between genes, proteins, and metabolites, facilitating the integration of multi-omics data [74].

Transfer learning involves leveraging pre-trained models on large datasets to improve performance on smaller task-specific datasets. This approach can be particularly useful for integrating multi-omics data, where pretrained models can provide valuable insights into underlying biological processes [75].

Explainable AI techniques aim to provide interpretable and transparent ML models. These methods can help researchers understand the decision-making process of ML models, ensuring that the results are biologically meaningful and trustworthy. Explainable artificial intelligence (AI) can enhance the interpretability of multi-omics data integration and guide the development of personalized interventions [76].

**Figure 1:**
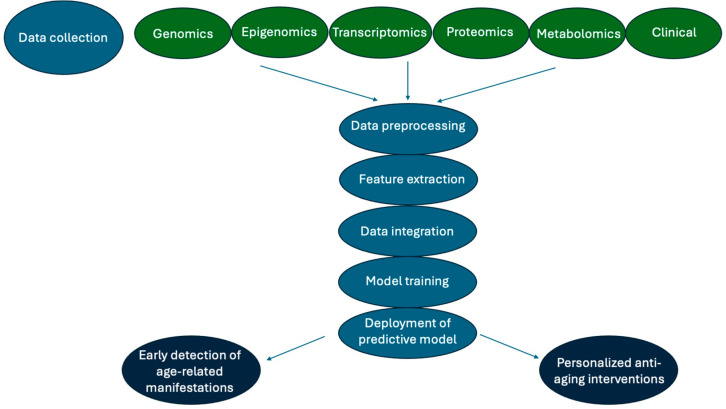
Workflow of multi-omics data integration with machine learning in aging research.

The workflow of multi-omics data integration with ML in aging research is illustrated in Figure 1.

A summary of ML techniques and their applications in multi-omics aging research is presented in Table 2.

### 3.4. Practical Guidance on Selecting Machine Learning Methods for Multi-Omics Research in Geroscience

Data from multi-omics technologies are often characterized by diversity and complexity, hindering analysis and drawing conclusions. When selecting ML methods for multi-omics integration in geroscience, it is crucial to consider the specific characteristics of the data and the research objectives. As a practical guide, we can apply the following ML methods for each of the following basic indications [20,77,78,79,80]:

- Genomic, epigenomic, and proteomic data analysis.
  - Random forests: ideal for telomere length analysis.
  - Support vector machines (SVMs): Suitable for distinguishing between benign and pathogenic mutations in genomic instability studies. Also ideal for identification of proteins with high aggregation potential.
  - Gradient boosting machines (GBMs): effective in identifying key epigenetic modifications that contribute to aging.
  - CNNs: best for analyzing proteomic data, particularly when dealing with structural data like protein imaging or spatial transcriptomics, where the spatial relationships between features are important.
  - Recurrent neural networks (RNNs) or long short-term memory (LSTM): Ideal for analyzing genomic sequences where the order of nucleotides (sequential data) is crucial. These models are particularly effective in understanding mutations that affect protein structure and function.
  - Fully connected neural networks (FCNNs): suitable for integrating multi-omics data (e.g., genomic, epigenomic, and proteomic) to classify complex age-related changes where high-dimensional data need to be processed.
- Longitudinal or dynamic data analysis.
  - RNNs or long short-term memory (LSTM): Particularly suited for analyzing changes in biomarkers over time. These networks are designed to handle sequential data, making them ideal for capturing temporal patterns in longitudinal data.
- Predictive models.
  - Ensemble methods: useful for robust predictive modeling by combining the strengths of multiple algorithms.
  - Multiple regression combined with ML techniques: offers a simpler interpretable approach to prediction when the focus is on a few key variables.
- Novel biomarker discovery.
  - CNNs: ideal when the biomarker discovery involves image data or spatially structured data, such as histopathological images or spatial transcriptomics data.
  - Autoencoders: A type of neural network ideal for unsupervised learning. Ideal when we have large complex multi-omics data and we want to find hidden patterns, making it easier to spot new biomarkers.
- Drug efficacy predictions and personalized medicine interventions.
  - GBMs.
- Modeling complex interactions among various age-related pathways (e.g., autophagy).
  - deep learning models, including GNNs for graph-based approaches.
- Explainable AI techniques: should be incorporated to ensure that the outcomes of ML models are interpretable and actionable, particularly in a clinical setting where the biological significance of the results must be clearly understood.

## 4. Case Studies and Applications

### 4.1. Predictive Modeling of Age-Related Diseases

ML models have been successfully applied to predict age-related diseases by integrating multi-omics data. These models can identify individuals at high risk and guide personalized interventions [48]. Some notable examples include Alzheimer’s disease (AD) and CVD.

AD is a neurodegenerative disorder characterized by cognitive decline and memory loss. Integrating genomic, transcriptomic, and proteomic data has led to the identification of biomarkers and predictive models for AD. For instance, ML models have been used to identify genetic variants and gene expression profiles associated with AD risk. Proteomic analyses have revealed changes in protein expression and post-translational modifications that correlate with disease progression. Integrating these datasets with clinical data has improved the accuracy of predictive models and facilitated early diagnosis and intervention [49]. AI and ML have also optimized diagnostic procedures and outperformed traditional neuropsychological tests in identifying cognitive impairment. For example, AI-driven mobile screening tests and game-based intelligence tests have demonstrated superior performance compared with conventional methods. Additionally, AI techniques have enhanced virtual reality assessments, offering high ecological validity and new avenues for cognitive rehabilitation [50]. A recent study proposed an ML model trained on a set of neuropsychological, neurophysiological, and clinical data to predict cognitive decline in MCI and AD patients. The study collected data from 4848 patients, including those diagnosed with AD and MCI. The results showed a diagnostic accuracy of 86%, with a sensitivity of 72% and a specificity of 91% for clinical data prediction with MMSE scores [51].

CVD is a leading cause of mortality worldwide. Integrating multi-omics data has provided valuable insights into the molecular mechanisms underlying CVD. For example, genomic data can identify genetic predispositions to CVD, while transcriptomic and proteomic data can reveal changes in gene and protein expression associated with disease states. Metabolomic profiling can provide information on metabolic shifts that contribute to CVD. ML models have been used to integrate these datasets, leading to the development of predictive models for CVD risk and progression. A recent study has utilized ML to investigate the presence of multiple factors in the risk of aortic stenosis. The findings indicated that significant features present in aortic stenosis patients included older age, arterial hypertension, aortic regurgitation, ascending aortic dilatation, and bicuspid aortic valve. These insights suggest that hypertension and other factors play a crucial role in the hemodynamic and anatomical progression of AS, highlighting the implications of ML for a comprehensive risk assessment and early intervention strategy [52].

### 4.2. Personalized Medicine Approaches for Aging Populations

#### 4.2.1. Genomic Instability Interventions

Targeting genomic instability involves enhancing DNA repair mechanisms and protecting against DNA damage. Several interventions have shown promise in preclinical models.

Poly(ADP-ribose) polymerase (PARP) inhibitors. These compounds enhance the efficacy of DNA repair by inhibiting PARP enzymes, which are involved in the repair of single-strand breaks. Studies in mice have demonstrated that PARP inhibitors can reduce DNA damage and extend lifespan. ML models can identify individuals with high levels of DNA damage or mutations that may benefit from PARP inhibitors by analyzing genomic and epigenomic data to pinpoint those most likely to respond positively [53].

NAD+ precursors. Nicotinamide riboside (NR) and nicotinamide mononucleotide (NMN) are precursors of NAD+, a coenzyme involved in DNA repair and cellular metabolism. Supplementation with NAD+ precursors has been shown to improve DNA repair capacity, reduce genomic instability, and extend lifespan in mice. ML can analyze metabolomic profiles to identify candidates with low NAD+ levels or impaired NAD+ metabolism who may benefit from such supplementation [54].

#### 4.2.2. Telomere Attrition Interventions

Telomere attrition can be addressed by enhancing telomerase activity or protecting telomeres from damage.

Telomerase activators. Compounds that activate telomerase, such as TA-65, have been shown to elongate telomeres and improve cellular function in aged mice. These interventions can delay the onset of age-related diseases and extend lifespan. ML models can predict telomere length and assess telomerase activity from genomic and proteomic data, identifying individuals who would benefit most from telomerase activators [81].

Telomere protective compounds. Certain small molecules and natural compounds, such as resveratrol and its derivatives, have been shown to protect telomeres from oxidative damage and improve telomere maintenance. These compounds can enhance telomere stability and promote healthy aging. By analyzing oxidative stress markers and telomere length data, ML can identify individuals with significant telomere attrition and oxidative damage who may benefit from telomere-protective compounds [55].

#### 4.2.3. Epigenetic Alterations Interventions

Targeting epigenetic alterations involves modulating DNA methylation and histone modifications to restore youthful gene expression patterns.

DNA methyltransferase (DNMT) inhibitors. Compounds that inhibit DNMTs, such as 5-azacytidine, can reduce aberrant DNA methylation and restore normal gene expression patterns. Preclinical studies have shown that DNMT inhibitors can improve metabolic health and extend lifespan in mice. ML algorithms can analyze epigenomic data to detect aberrant methylation patterns and predict which individuals might benefit from DNMT inhibitors [56].

Histone deacetylase (HDAC) inhibitors. HDAC inhibitors, such as suberoylanilide hydroxamic acid (SAHA), can enhance histone acetylation and promote a more open chromatin structure. These compounds have been shown to improve cognitive function and extend lifespan in animal models. ML models can predict beneficial epigenetic changes and identify candidates with histone modification profiles indicative of age-related epigenetic alterations [57].

#### 4.2.4. Loss of Proteostasis Interventions

Restoring proteostasis involves enhancing protein quality control systems and promoting the degradation of misfolded proteins.

Chaperone activators. Small molecules that activate heat shock proteins (HSPs), such as geldanamycin, can enhance protein folding and reduce the accumulation of misfolded proteins. Preclinical studies have demonstrated that chaperone activators can improve proteostasis and extend lifespan in mice. ML can analyze proteomic data to identify individuals with high levels of misfolded proteins who might benefit from chaperone activators [21].

Proteasome activators. In addition to chaperone activators, proteasome activators represent another promising avenue for restoring proteostasis in aging cells. The proteasome is a complex protein assembly responsible for degrading damaged or misfolded proteins tagged with ubiquitin, a process vital for maintaining cellular function and preventing disease. Enhancing proteasome activity has been shown to alleviate proteotoxic stress and improve cellular resilience against aging-related dysfunctions. For example, studies have demonstrated that compounds like bortezomib, originally used in cancer therapy to induce proteasome inhibition, can be tuned to subtly activate proteasome activity, thus promoting the clearance of toxic proteins in neurodegenerative disease models [58]. Implications of ML can significantly augment the development and application of proteasome activators in aging research. By analyzing vast datasets of proteomic profiles, ML algorithms can identify patterns indicating proteasome inefficiency and predict which individuals or models might benefit most from targeted proteasome activation. Furthermore, ML can assist in screening for novel proteasome activators by predicting their efficacy and safety based on structural and functional data. This predictive capability is crucial for optimizing therapeutic strategies and tailoring interventions to specific aging populations or disease phenotypes, thus embodying the principles of personalized medicine.

#### 4.2.5. Disabled Macroautophagy Interventions

Enhancing macroautophagy involves stimulating the autophagic machinery to clear damaged cellular components.

mTOR inhibitors. Compounds that enhance autophagy, such as mTOR inhibitors, can promote the degradation of damaged proteins and organelles. Preclinical studies have shown that mTOR inhibition can enhance autophagy, improve metabolic health, reduce protein aggregation, and extend lifespan in mice. ML models can assess autophagic activity from multi-omics data and identify those with impaired autophagy who could benefit from these enhancers [59]. Furthermore, ML can predict mTOR pathway activity by analyzing transcriptomic and proteomic data and identifying individuals with overactive mTOR signaling who might benefit from mTOR inhibitors [60].

AMPK activators. Metformin and other AMPK activators stimulate autophagy by activating the energy-sensing AMPK pathway. These compounds have been shown to improve insulin sensitivity, reduce inflammation, and extend lifespan in animal models. ML models can integrate metabolomic and transcriptomic data to identify individuals with low AMPK activity who might benefit from AMPK activators [61].

#### 4.2.6. Deregulated Nutrient Sensing Interventions

Modulating nutrient sensing pathways involves targeting key regulators such as mTOR, AMPK, and sirtuins.

mTOR inhibitors. As mentioned earlier, rapamycin and its analogs inhibit mTOR signaling, which can mimic the effects of caloric restriction and promote longevity. Preclinical studies have demonstrated that mTOR inhibitors can extend lifespan and improve metabolic health in various animal models. ML can identify individuals with overactive mTOR signaling through integrated omics data analysis, suggesting those who may benefit from such interventions [30].

Sirtuin activators. Resveratrol and other sirtuin activators enhance the activity of sirtuins, a family of NAD+-dependent deacetylases involved in metabolic regulation and stress resistance. These compounds have been shown to improve mitochondrial function, enhance stress resistance, and extend lifespan in animal models. ML can analyze sirtuin activity from proteomic and metabolomic data to identify candidates who would benefit from sirtuin activators [62].

#### 4.2.7. Mitochondrial Dysfunction Interventions

Improving mitochondrial function involves enhancing mitochondrial biogenesis and protecting against oxidative damage.

Mitochondrial-targeted antioxidants. Compounds such as MitoQ and SkQ1 selectively target mitochondria to reduce oxidative stress. Preclinical studies have shown that mitochondrial-targeted antioxidants can improve mitochondrial function, reduce ROS production, and extend lifespan in animal models. ML can predict mitochondrial dysfunction by analyzing mtDNA mutations and oxidative stress markers, identifying individuals who might benefit from mitochondrial-targeted antioxidants [63].

PPARγ coactivator 1α (PGC-1α) activators. PGC-1α is a key regulator of mitochondrial biogenesis. Compounds that activate PGC-1α, such as bezafibrate, have been shown to enhance mitochondrial function and extend lifespan in mice. ML can assess PGC-1α activity from transcriptomic and proteomic data to identify candidates with impaired mitochondrial biogenesis who might benefit from these activators [64].

#### 4.2.8. Cellular Senescence Interventions

Targeting cellular senescence involves selectively eliminating senescent cells or modulating the senescence-associated secretory phenotype (SASP).

Senolytics. Compounds such as dasatinib and quercetin selectively induce apoptosis in senescent cells. Preclinical studies have shown that senolytics can reduce the burden of senescent cells, improve tissue function, and extend lifespan in mice. ML can identify biomarkers of cellular senescence from multi-omics data, pinpointing individuals who would benefit from senolytic therapies [65].

SASP inhibitors. JAK inhibitors, such as ruxolitinib, can modulate the pro-inflammatory secretome of senescent cells. These compounds have been shown to reduce chronic inflammation, improve tissue function, and extend lifespan in animal models. ML can predict the SASP profile from transcriptomic and proteomic data, identifying those with high SASP activity who might benefit from SASP inhibitors [66].

#### 4.2.9. Stem Cell Exhaustion Interventions

Rejuvenating stem cells involves enhancing stem cell function and promoting their self-renewal capacity.

Wnt pathway modulators. Activation of the Wnt signaling pathway has been shown to enhance stem cell self-renewal and improve tissue regeneration. Compounds such as R-spondin1 have been shown to rejuvenate aged stem cells and extend lifespan in mice. ML can analyze stem cell markers and Wnt signaling activity from multi-omics data to identify candidates who might benefit from Wnt pathway modulators [37].

Young blood factors. Transfusion of blood from young to old animals has been shown to rejuvenate aged tissues and improve stem cell function. Factors such as GDF11 and TIMP2 have been identified as potential mediators of these rejuvenating effects. ML can predict the benefits of young blood factors by analyzing circulating biomarkers and stem cell function data, and identifying individuals who might benefit from such interventions [67].

#### 4.2.10. Altered Intercellular Communication Interventions

Restoring normal intercellular communication involves modulating signaling pathways and improving immune function.

Cytokine modulators. IL-6 inhibitors, such as tocilizumab, can reduce pro-inflammatory signaling and improve immune function. Preclinical studies have shown that cytokine modulators can reduce chronic inflammation and extend lifespan in animal models. ML can analyze cytokine profiles and immune function data to identify individuals with dysregulated intercellular communication who might benefit from cytokine modulators [82].

Immune rejuvenation. Interventions that rejuvenate the immune system, such as thymic regeneration or the transplantation of young immune cells, have been shown to improve immune function and extend lifespan in animal models. ML can predict immune system aging from multi-omics data, identifying candidates for immune rejuvenation therapies [83].

#### 4.2.11. Chronic Inflammation Interventions

Reducing chronic inflammation involves targeting pro-inflammatory pathways and enhancing anti-inflammatory signaling.

NF-κB inhibitors. Compounds that inhibit the NF-κB pathway, such as aspirin and salicylates, can reduce chronic inflammation and improve metabolic health. Preclinical studies have demonstrated that NF-κB inhibitors can extend lifespan and improve health span in animal models. ML can identify individuals with high NF-κB activity from transcriptomic and proteomic data, suggesting those who might benefit from NF-κB inhibitors [84].

Anti-inflammatory cytokines. IL-10 and other anti-inflammatory cytokines can modulate the immune response and reduce chronic inflammation. These cytokines have been shown to improve tissue function and extend lifespan in preclinical studies. ML can predict the benefits of anti-inflammatory cytokines by analyzing inflammatory markers and immune function data and identifying individuals who might benefit from these therapies [85].

#### 4.2.12. Dysbiosis Interventions

Restoring a healthy microbiome involves modulating the composition and function of the gut microbiota.

Probiotics. The administration of beneficial bacteria, such as Lactobacillus and Bifidobacterium, can improve gut health and metabolic function. Preclinical studies have shown that probiotics can extend lifespan and improve health span in animal models. ML can analyze microbiome composition and metabolic profiles to identify individuals with dysbiosis who might benefit from probiotic interventions [46].

Fecal microbiota transplantation (FMT). Transplanting gut microbiota from young to old animals has been shown to rejuvenate the gut microbiome and improve metabolic health. Preclinical studies have demonstrated that FMT can extend lifespan and improve health span in animal models. ML can predict the benefits of FMT by analyzing microbiome diversity and function data and identifying individuals who might benefit from this intervention [86].

### 4.3. Identification of Novel Biomarkers

The discovery of novel biomarkers is crucial for early diagnosis and treatment of age-related diseases. ML techniques can be used to analyze multi-omics data and identify potential biomarkers. These biomarkers can then be validated and their efficacy predicted using ML models, providing valuable tools for clinical applications.

#### 4.3.1. Biomarker Discovery in Neurodegenerative Diseases

Neurodegenerative diseases, such as Parkinson’s disease and amyotrophic lateral sclerosis, are characterized by progressive neuronal loss and functional decline. Identifying biomarkers for early diagnosis and monitoring disease progression is challenging due to the complex and multifactorial nature of these diseases. Integrating multi-omics data, including genomic, transcriptomic, proteomic, and metabolomic profiles, can reveal molecular changes associated with neurodegeneration. ML models can identify key biomarkers that distinguish between different disease stages and predict disease progression, facilitating early intervention and personalized treatment [48].

#### 4.3.2. Biomarker Discovery in Inflammatory Diseases

Inflammatory diseases, such as rheumatoid arthritis and inflammatory bowel disease, involve dysregulated immune responses and chronic inflammation. Identifying biomarkers for early diagnosis and monitoring disease activity is essential for effective treatment. Integrating multi-omics data, including genomic, transcriptomic, proteomic, and metabolomic profiles, can reveal molecular changes associated with inflammation. ML models can identify key biomarkers that predict disease activity and treatment response, guiding personalized interventions and improving patient outcomes [87].

#### 4.3.3. Biomarker Discovery in Reproductive Aging

Recent studies have leveraged multi-omics approaches to identify potential biomarkers for ovarian aging. One notable discovery is the multifunctional protein secreted phosphoprotein 1 (SPP1), also known as osteopontin. This protein has been implicated in various biological processes, including inflammation, immune responses, and tissue remodeling. However, its role in ovarian aging has not been fully explored until now. Spatial transcriptomic analyses of mouse ovaries have revealed a significant decline in SPP1 expression in aging tissues compared with younger ones. Furthermore, single-cell RNA sequencing highlighted associations between SPP1 and key genes such as ITGAV, ITGB1, CD44, MMP3, and FN1, indicating its regulatory role in the ovarian microenvironment. Additionally, data from the Human Protein Atlas showed a marked decrease in SPP1 levels in the ovaries of older women (aged 40–49) compared with younger women (aged 20–29). These findings suggest that SPP1 could serve as a valuable biomarker for ovarian aging, aiding in the early diagnosis and personalized treatment of age-related reproductive conditions. The decline in SPP1 expression with age underscores its potential utility in developing targeted therapies aimed at enhancing reproductive health and managing ovarian aging more effectively [88].

#### 4.3.4. Biomarker Discovery in Cancer Research

Recent advancements in multi-omics and ML have also enhanced biomarker identification for malignancies, including non-small-cell lung cancer. A notable study integrated RNA-Seq, miRNA-Seq, copy number variation, and DNA methylation data with whole-slide imaging data to identify potential biomarkers.

ML models were trained on each data type independently and their outputs were combined using a late fusion approach, significantly improving diagnostic accuracy. This method provided robust biomarkers that effectively distinguished between NSCLC subtypes, demonstrating high-performance metrics like F1 score and AUC. The study also highlighted the potential for integrating these biomarkers into clinical decision support systems, enhancing personalized diagnostic and treatment strategies for lung cancer [89].

### 4.4. Applications of Machine Learning Algorithms in Medical Diagnosis

Recent developments in ML techniques have shown significant promise in enhancing the analysis and integration of multi-omics data, particularly in medical diagnostics. For example, a novel approach discussed in the recent literature involves a hybrid model that combines CNNs with a pruned ensemble of extreme learning machines (ELMs). This model, designed for breast cancer detection and diagnostics, leverages the strengths of both deep learning and ML to improve image analysis accuracy to 86%, surpassing traditional methods. By integrating CNN for robust feature extraction and ELM for efficient classification, this model exemplifies the potential of hybrid ML systems in not only enhancing diagnostic accuracy but also in facilitating early disease detection, which is critical in treatment planning and outcome improvement [90].

Additionally, AI is increasingly playing a transformative role in cardiology, enabling enhanced diagnostic and predictive capabilities. ML algorithms, particularly neural networks, have been applied successfully in interpreting complex cardiovascular imaging data, leading to improved diagnostic accuracy in conditions such as coronary artery disease, hypertrophic cardiomyopathy, and atrial fibrillation. These AI-driven tools assist clinicians by providing more precise and rapid interpretations of echocardiograms and electrocardiograms, potentially leading to better patient outcomes by enabling timely and accurate treatment decisions [91]. Specifically, a recent study has explored the application of ML in predicting atrial fibrillation in patients with embolic strokes of undetermined sources (ESUS), showcasing another promising dimension of AI in cardiovascular health diagnostics. A study utilized ML models, including SVMs, multilayer perceptron (MLP), XGBoost, and random forest, to analyze clinical and echocardiographic data of 157 ESUS patients. The SVM model exhibited the highest efficacy, with an area under the curve (AUC) of approximately 0.736, demonstrating a robust capability to predict AF occurrences [92]. Another study focused on predicting myocardial ischemia by integrating exploratory data analysis and ML models, achieving accuracies above 80% [93].

Furthermore, recent advancements in ML have significantly enhanced prostate cancer diagnostics, particularly through the use of radiomics and AI in molecular imaging. By applying data-characterization algorithms to positron emission tomography scans, radiomics helps to accurately distinguish between pathological and physiological tracer uptakes. ML models, including CNNs, can analyze these radiomics features to predict Gleason scores, influencing treatment decisions and enhancing personalized patient management in oncology [94].

Such innovative approaches highlight the transformative impact of advanced ML techniques in medical diagnosis, offering new pathways for the effective analysis of complex biological datasets.

## 5. Challenges and Limitations

### 5.1. Data Availability, Integration, and Computational Complexity

A limitation of integrating ML with multi-omics technologies is the assumption of readily available large well-annotated omics datasets. In the real world, such comprehensive datasets may be scarce, particularly in underfunded research environments or regions with limited technological infrastructure. Even in the presence of such data, integrating diverse datasets from different omics layers presents significant computational challenges. Advanced computational models and algorithms are required to handle the complexity of multi-omics data integration. Additionally, the development of user-friendly tools and platforms is essential to facilitate the application of these models in biomedical research [95].

### 5.2. Data Heterogeneity

Biomedical data come from various sources and formats, such as genomic sequences, imaging data, and electronic health records. Integrating these diverse datasets requires sophisticated data preprocessing and normalization techniques. For example, genomic data may need to be aligned and variant-called, while imaging data may require segmentation and feature extraction [95].

### 5.3. Data Quality

Biomedical data can be noisy, incomplete, or biased. Ensuring data quality is crucial for the development of accurate and reliable ML models. Data preprocessing steps, such as normalization, imputation, and outlier detection, are essential for improving data quality. Additionally, techniques like cross-validation and bootstrapping can help assess model robustness and reliability [96].

### 5.4. Ethical Considerations and Data Privacy

The use of multi-omics data in biomedical research raises several ethical considerations and data privacy concerns.

#### 5.4.1. Informed Consent

Researchers must obtain informed consent from participants for the collection, storage, and analysis of their biological samples and data. Participants should be informed about the purpose of the study, the types of data being collected, and the potential risks and benefits [97].

#### 5.4.2. Data Privacy and Security

Protecting the privacy and confidentiality of participants’ data is paramount. Strategies to ensure data privacy include data anonymization, secure data storage, and controlled access to data. Implementing robust data security measures, such as encryption and secure data transfer protocols, is also essential [98].

#### 5.4.3. Ethical Use of Data

Researchers must ensure that the data are used ethically and responsibly. This includes avoiding misuse or misinterpretation of data, maintaining transparency in data analysis and reporting, and addressing potential biases in data collection and analysis [99].

#### 5.4.4. Equity and Inclusion

Ensuring that diverse populations are represented in biomedical research is critical for generalizability and equity. Researchers should strive to include participants from different demographic backgrounds, including age, gender, ethnicity, and socioeconomic status, to capture a comprehensive understanding of biological processes and their implications for different populations [100].

### 5.5. Limitations of Current Studies

Current studies on aging often have limitations, such as small sample sizes, lack of longitudinal data, and potential biases. Small sample sizes can limit the statistical power and generalizability of study findings. Collaborative efforts to pool data from multiple studies can help increase sample sizes and improve the robustness of results. Longitudinal data are essential for studying the dynamic processes of aging and disease progression. However, collecting longitudinal data can be challenging due to the time and resources required. Developing standardized protocols for longitudinal data collection and analysis can help address this challenge. Potential biases in data collection and analysis can impact the validity of study findings. Addressing these biases requires careful consideration of study design, data collection methods, and statistical analysis techniques. Ensuring diversity and inclusion in study populations can also help mitigate biases.

Addressing these limitations requires collaborative efforts in data sharing, the inclusion of diverse populations in research, and the development of standardized protocols for data collection and analysis [95].

## 6. Future Directions

### 6.1. Advances in Omics Technologies

Emerging omics technologies, such as single-cell omics and spatial transcriptomics, have the potential to provide unprecedented insights into the aging process. These technologies can capture molecular changes at higher resolution and across different tissues, offering new opportunities for aging research.

#### 6.1.1. Single-Cell Omics

Single-cell genomics and transcriptomics, including single-cell RNA sequencing (scRNA-seq) and single-cell ATAC sequencing (scATAC-seq), enable the analysis of gene expression and chromatin accessibility at the single-cell level. Single-cell proteomics can analyze protein levels and post-translational modifications in individual aging cells. These technologies can reveal cellular heterogeneity and identify rare cell populations involved in aging and age-related diseases. Integrating single-cell omics data with other omics layers can provide a comprehensive view of the molecular mechanisms underlying aging [101].

#### 6.1.2. Spatial Omics

This approach can map gene and protein expression in tissues, maintaining the spatial context that is crucial for understanding the complex cellular interactions during aging. It could lead to discoveries about how tissue microenvironments change with age and contribute to senescence or regeneration [102].

### 6.2. Development of Sophisticated Machine Learning Models

Innovations in ML, such as deep learning and explainable AI, can further enhance the analysis of multi-omics data. Collaborative efforts in data sharing and open science will be crucial for advancing the field and translating research findings into clinical applications.

#### 6.2.1. Deep Learning

Deep learning techniques, such as convolutional neural networks and recurrent neural networks, have shown promise in analyzing complex biomedical data. These models can automatically learn hierarchical representations from raw data, making them suitable for integrating multi-omics data. Neural networks can model complex patterns and interactions within large-scale omics data, potentially identifying new biomarkers of aging or targets for drug development [71].

#### 6.2.2. Explainable AI

Explainable AI techniques aim to provide interpretable and transparent ML models. These methods can help researchers understand the decision-making process of ML models, ensuring that the results are biologically meaningful and trustworthy. Explainable AI can enhance the interpretability of multi-omics data integration and guide the development of personalized interventions [76]. This is particularly important in precision medicine, where the ability to interpret and trust AI models can significantly impact patient outcomes. Explainable AI not only supports the integration of AI with digital health data but also helps in improving the accuracy and reliability of predictive models, ensuring that the results are actionable and meaningful for clinical practice [103].

### 6.3. Implementation in Personalized Medicine

The hallmark-targeted interventions discussed above hold significant promise for future personalized medicine approaches. These approaches targeting the hallmarks of aging hold the potential to revolutionize aging research and extend health span and lifespan. Implementing these personalized interventions requires advanced computational tools and ML models to analyze and integrate multi-omics data, predict individual responses to treatments, and optimize therapeutic strategies. ML can provide insights into the molecular profiles of patients, identify potential candidates for specific interventions, and continuously refine treatment plans based on ongoing data collection and analysis. Specifically, by integrating ML techniques with multi-omics data, healthcare providers can perform the following:

- Identify biomarkers (e.g., proteins, RNAs, and metabolites) and genetic risk factors that predict the onset of age-related diseases (e.g., Alzheimer’s or CVD) well before clinical symptoms manifest.
- Explore how changes in metabolites and gut microbiota influence aging and the risk of diseases, potentially leading to dietary recommendations, supplementation, or probiotic treatments to maintain health and longevity.
- Develop individualized treatment plans based on a person’s omics profile to maximize efficacy and minimize side effects, particularly for complex diseases like cancer or neurodegenerative diseases.
- Develop novel treatments that target the specific molecular mechanisms driving aging in each patient, promoting healthy aging and extending life expectancy. For example, a patient with significant telomere attrition might benefit from telomerase activators, telomere protective compounds, or editing of the genes affecting telomere length with CRISPR-CAS9. Another patient with mitochondrial dysfunction could receive mitochondrial-targeted antioxidants and PGC-1α activators.

By leveraging the power of multi-omics data and ML, we can develop precise individualized treatments that address the unique needs of each aging individual, paving the way for a future where healthy aging is achievable for all.

**Figure 2:**
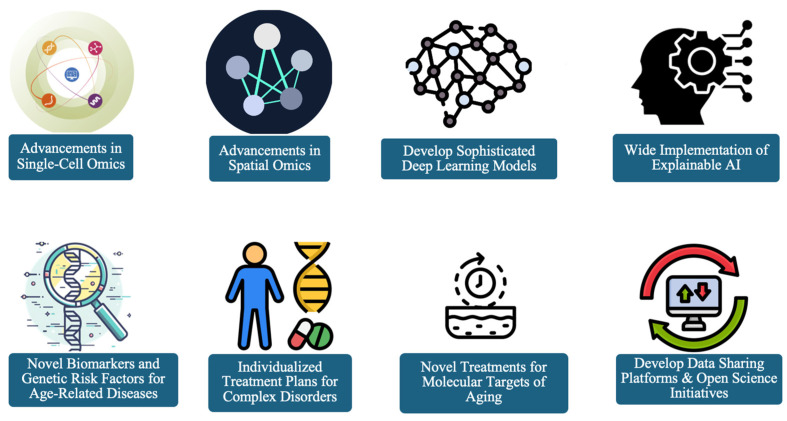
Future directions in the integration of machine learning with multi-omics technologies.

The future directions arising from the integration of ML with multi-omics technologies are illustrated in Figure 2.

### 6.4. Collaborative Efforts in Data Sharing and Open Science

Collaborative efforts in data sharing and open science are essential for advancing aging research. Sharing data and resources can facilitate the replication and validation of findings, improve the robustness of results, and accelerate the translation of research into clinical applications.

#### 6.4.1. Data Sharing Platforms

Developing data-sharing platforms that enable researchers to share multi-omics data and analytical tools can promote collaboration and reproducibility. These platforms should ensure data privacy and security, provide standardized protocols for data sharing, and support interoperability between different datasets [104].

#### 6.4.2. Open Science Initiatives

Open science initiatives that promote transparency, accessibility, and reproducibility in research can enhance the credibility and impact of aging research. These initiatives include open access to publications, open data repositories, and collaborative research networks. Supporting open science can facilitate the translation of research findings into clinical applications and promote the development of personalized interventions for aging populations [105].

## 7. Conclusions

The integration of multi-omics data with ML offers a powerful approach to delve into a better understanding of the molecular hallmarks of aging. Advances in these technologies can have a significant impact on aging research. This approach has the potential to identify critical biomarkers and therapeutic targets, paving the way for personalized interventions aimed at extending both health span and lifespan. The future of aging research lies in the seamless integration of these technologies, supported by collaborative efforts in data sharing and open science. Such advancements will be essential in overcoming current challenges and translating research findings into clinical practice. Ultimately, by leveraging the power of multi-omics data and ML, we can move closer to a future where healthy aging is achievable for all.

## Figures and Tables

**Table 1:** Summary of aging hallmarks, their molecular mechanisms, and potential interventions.

Aging Hallmark	Molecular Mechanisms	Examples of Interventions	References
Genomic Instability	Increased frequency of mutations and structural alterations in DNA.	PARP inhibitors (e.g., olaparib). NAD+ precursors (e.g., nicotinamide riboside). Antioxidants (e.g., glutathione).	[2,10,11,12,13,48,49]
Telomere Attrition	Telomere shortening due to end-replication problem and limited telomerase expression.	Telomerase activators (e.g., TA-65). Telomere protective compounds (e.g., resveratrol).	[2,15,16,50,51]
Epigenetic Alterations	Changes in DNA methylation, histone modifications, and chromatin remodeling.	HDAC inhibitors (e.g., suberoylanilide hydroxamic acid). DNMT inhibitors (e.g., 5-azacytidine).	[2,18,19,52,53]
Loss of Proteostasis	Accumulation of misfolded and aggregated proteins.	Chaperone activators (e.g., geldanamycin). Proteasome activators (e.g., spermidine).	[2,21,22,23,54]
Disabled Macroautophagy	Impairment of autophagy leads to the accumulation of damaged cellular components.	mTOR inhibitors (e.g., rapamycin). AMPK activators (e.g., metformin).	[2,25,26,55,56]
Deregulated Nutrient Sensing	Deregulation of insulin/IGF-1 signaling, mTOR, AMPK, and sirtuin pathways.	mTOR inhibitors (e.g., rapamycin). Sirtuin activators (e.g., resveratrol).	[2,28,29,57]
Mitochondrial Dysfunction	Impaired energy production, increased ROS, and mtDNA mutations.	Mitochondrial-targeted antioxidants (e.g., MitoQ). PGC-1α activators (e.g., bezafibrate).	[2,31,32,58,59]
Cellular Senescence	Permanent cell cycle arrest due to DNA damage, oxidative stress, etc.	Senolytics (e.g., dasatinib, quercetin). SASP inhibitors (e.g., ruxolitinib).	[2,34,35,36,60,61]
Stem Cell Exhaustion	Reduction in the number and function of stem cells.	Wnt pathway modulators (e.g., R-spondin1). Young blood factors (e.g., GDF-11, TIMP-2).	[2,37,38,62]
Altered Intercellular Communication	Changes in cell signaling and communication.	Cytokine modulators (e.g., tofacitinib). Immune rejuvenation therapies	[2,40,41,63,64]
Chronic Inflammation	Persistent low-grade inflammation is linked to various age-related diseases.	NF-κB inhibitors (e.g., aspirin). Anti-inflammatory cytokines (e.g., IL-10).	[2,43,44,65,66]
Dysbiosis	Changes in the composition and function of the microbiome.	Probiotics (e.g., *Lactobacillus* spp.). Fecal microbiota transplantation.	[2,46,47,67]

Abbreviations. AMPK (AMP-activated protein kinase); DNA (deoxyribonucleic acid); DNMT (DNA methyltransferase); GDF-11 (growth differentiation factor-11); HDAC (histone deacetylase); IGF-1 (insulin-like growth factor 1); IL-10 (interleukin-10); mTOR (mechanistic target of rapamycin); NAD+ (nicotinamide adenine dinucleotide); NF-κB (nuclear factor kappa-light-chain-enhancer of activated B cells); PARP (poly(ADP-ribose) polymerase); PGC-1α (peroxisome proliferator-activated receptor gamma coactivator 1-alpha); ROS (reactive oxygen species); SASP (senescence-associated secretory phenotype); TA-65 (telomerase activator-65); TIMP-2 (tissue inhibitor of metalloproteinases-2); Wnt (wingless/integrated).

**Table 2:** Summary of machine learning techniques and their applications in multi-omics aging research.

ML Technique	Description	Applications
Supervised Learning	Algorithms that learn from labeled data to make predictions.	Predicting genomic instability. Classifying mutations as benign or pathogenic. Predicting biological age. Identifying aging biomarkers. Disease risk prediction.
Unsupervised Learning	Algorithms that identify patterns in unlabeled data.	Discovering new age-associated biomarkers. Identifying distinct epigenetic signatures. Clustering age-related gene expression profiles and proteomic changes. Grouping proteins based on stability.
Reinforcement Learning	Algorithms that learn by interacting with an environment to maximize cumulative rewards.	Optimizing intervention strategies. Personalized treatment plans.
Deep Learning	Neural networks with multiple layers can model complex relationships in data.	Integrating multi-omics data, predicting age-related disease progression. Learning hierarchical representations from raw data. Improving predictive accuracy for age-related diseases.
Graph Neural Networks	Models that capture relationships and interactions between entities in a graph structure.	Modeling interactions between genes, proteins, and metabolites. Integrating multi-omics data for comprehensive analysis.
Convolutional Neural Networks	Deep learning models are particularly effective for analyzing spatial and visual data.	Integrating and analyzing imaging data. Detecting patterns in omics data.
Transfer Learning	Leveraging pretrained models to improve performance on new smaller datasets.	Enhancing model accuracy by leveraging pre-trained models. Transferring knowledge across different datasets.
Explainable Artificial Intelligence	Techniques to make artificial intelligence models’ decisions interpretable and transparent.	Providing interpretable and transparent machine learning models. Ensuring results are biologically meaningful, enhancing trust and understanding of machine learning predictions.

## Data Availability

No new data were created or analyzed in this study. Data sharing is not applicable to this article.
